# Natural Rubber-TiO_2_ Nanocomposite Film for Triboelectric Nanogenerator Application

**DOI:** 10.3390/polym13132213

**Published:** 2021-07-05

**Authors:** Weeraya Bunriw, Viyada Harnchana, Chalathorn Chanthad, Van Ngoc Huynh

**Affiliations:** 1 Materials Science and Nanotechnology Program, Faculty of Science, Khon Kaen University, Khon Kaen 40002, Thailand; weeraya_b@kkumail.com; 2 Department of Physics, Khon Kaen University, Khon Kaen 40002, Thailand; 3 Institute of Nanomaterials Research and Innovation for Energy (IN-RIE), NANOTEC-KKU RNN on Nanomaterials Research and Innovation for Energy, Khon Kaen University, Khon Kaen 40002, Thailand; 4 National Nanotechnology Center (NANOTEC), NSTDA, 111 Thailand Science Park, Paholyothin Road, Klong Luang, Pathum Thani 12120, Thailand; chalathorn@nanotec.or.th; 5 DTU Bioengineering, Department of Biotechnology and Biomedicine, Technical University of Denmark, 2800 Kongens Lyngby, Denmark; vannh@dtu.dk

**Keywords:** natural rubber, triboelectric nanogenerator, TiO_2_ nanoparticles, dielectric constant

## Abstract

In this research, natural rubber (NR)-TiO_2_ nanocomposites were developed for triboelectric nanogenerator (TENG) application to harvest mechanical energy into electrical energy. Rutile TiO_2_ nanoparticles were used as fillers in NR material to improve dielectric properties so as to enhance the energy conversion performance of the NR composite TENG. The effect of filler concentration on TENG performance of the NR-TiO_2_ composites was investigated. In addition, ball-milling method was employed to reduce the agglomeration of TiO_2_ nanoparticles in order to improve their dispersion in the NR film. It was found that the TENG performance was significantly enhanced due to the increased dielectric constant of the NR-TiO_2_ composite films fabricated from the ball-milled TiO_2_. The TENG, fabricated from the NR-TiO_2_ composite using 24 h ball-milled TiO_2_ at 0.5%wt, delivered the highest power density of 237 mW/m^2^, which was almost four times higher than that of pristine NR TENG. Furthermore, the applications of the fabricated NR-TiO_2_ TENG as a power source to operate portable electronics devices were also demonstrated.

## 1. Introduction

Energy harvesting technologies have attracted great attention because of the significance in producing sustainable energy sources to overcome energy crisis and climate change. In addition, the rapidly increasing number of personal electronic devices and other components for the Internet of Things (IoT) platform leads to the increasing demand for energy. Triboelectric nanogenerator (TENG) is a mechanical energy harvesting device based on the combination of contact electrification and electrostatic induction effects [1]. TENG has gained much interest due to its high energy conversion efficiency with high power output, straightforward fabrication process, and low cost [2]. Apart from energy harvesting applications, TENGs also have the potential to be used for many self-powered sensor applications, including physical, chemical, gas, and liquid sensors [3,4,5,6].

A wide range of materials can be used to fabricate TENG; most of them are polymeric materials [7]. The common known materials are polytetrafluoroethylene (PTFE) [8,9], polydimethylsiloxane (PDMS) [10,11], polyvinylidenefluoride (PVDF) [12,13], and polymethyl methacrylate (PMMA) [14,15]. Natural rubber (NR) or polyisoprene is one of the natural polymers with good flexibility and strength employed in a wide range of applications [16]. Most of NR products, such as car tires, gloves, shoe insoles, and mattresses, involve the applications in direct contact with mechanical energy sources. NR is one of the triboelectric materials located in the triboelectric series possessing slightly negative polarity [7]. In this regard, the fabrication of NR-based TENGs would be beneficial for boosting power output to realize practical applications of the TENG.

Generally, the output performance of TENG is a function of triboelectric charges on triboelectric materials which depend on electrification between two triboelectric materials, surface area, and ability of surface to hold charges [17,18]. In order to improve triboelectric charge density on the surface, many approaches have been proposed, including surface patterning with nanostructures [19,20] and improving dielectric properties of triboelectric materials [21,22,23,24]. For the latter case, filling nanomaterials, such as SiO_2_, TiO_2_, BaTiO_3_, and SrTiO_3_ in polymer triboelectric materials, were reported to improve dielectric constant and TENG performance [23]. Among these filler materials, TiO_2_ is an extensively used material for a wide range of applications due to many excellent physical and chemical properties, including optical-electronics [25], photocatalytic properties[26], chemically stability, nontoxicity, as well as low cost. TiO_2_ exists in three main polymorph phases including anatase, brookite, and rutile [27]. Among them, rutile-TiO_2_ exists as the most thermodynamically stable phase and exhibits a high dielectric constant [28,29]. 

In this work, rutile-TiO_2_ nanoparticles were incorporated into NR material forming NR-TiO_2_ composite film which was then used as a triboelectric material to convert mechanical energy into electricity. However, the NR-TiO_2_ composite fabricated by mixing the TiO_2_ nanoparticles directly with NR latex did not greatly improve the TENG output, possibly due to the agglomeration of as-received TiO_2_ nanoparticles. In the present work, the ball-milling method is proposed as an effective and straightforward method to alleviate the agglomeration of TiO_2_ nanoparticles prior to mixing with NR latex, thereby improving the dispersion of nanoparticles in the NR matrix. The effects of milling times and the concentration of TiO_2_ nanoparticles in the NR film on dielectric properties and TENG output performance were investigated. The performance of the NR-TiO_2_ TENG was probed under a vertical contact-separation mode. The morphologies and dielectric properties of the composite films were examined using a scanning electron microscope (SEM) and an impedance analyzer, respectively.

## 2. Materials and Methods

### 2.1. Preparation of NR-TiO_2_ Composite Films

The commercial NR latex (purchased from the Thai Rubber Latex Group Public Co., Ltd., Samut Prakan, Thailand) with a dry rubber content of 61% and rutile TiO_2_ nanoparticles (Briture Co., Ltd., Hefei, China) were used in this work. NR latex and the as-received TiO_2_ nanoparticles at 0.1, 0.2, 0.3, 0.4, and 0.5%wt were mixed by magnetic stirring for 5 min to ensure a homogeneous mixing. Then, 2 mL of the mixture was cast on an FTO substrate (Bangkok Solar Power Co., Ltd., Chachoengsao, Thailand) with an area of 4 cm × 4 cm so as to control the film thicknesses of approximately 0.5 mm. Three samples were prepared for each of the experimental conditions. The cast samples were then left to dry at room temperature for 4 days and cured at 80 °C for 2 h. In the present work, low curing temperature with long curing durations were employed in order to control the uniformity of the film top surface. Then, the samples were tested for the TENG performance, as described in Section 2.3. 

In addition, the TiO_2_ nanoparticles were dry ball-milled prior to mixing with NR latex. Yttria-stabilized zirconia balls and TiO_2_ nanoparticles were put in a polyethylene (PE) plastic vial at the ball to a powder weight ratio (BPR) of 4:1. The ball-milling process was performed at a milling speed of 250 rpm for 6, 12, and 24 h. The ball-milled TiO_2_ nanoparticles were then incorporated to NR latex following the same procedure, as described above. The composite films with ball-milled TiO_2_ for 6, 12, and 24 h were labeled as “NR-TiO_2_-B6h, NR-TiO_2_-B12h, and NR-TiO_2_-B24h”, respectively.

### 2.2. Material Characterizations

The morphologies and crystal structure of the composite films were investigated using a SEM (FEI, Helios Nanolab, Waltham, MA, USA) and an X-ray diffraction technique (XRD) (PANalytical EMPYREAN, Malvern, UK), respectively. Dielectric constants were measured using an impedance analyzer (Keysight, E4990A, Colorado Springs, CO, USA) at room temperature. Chemical functional group analysis was performed using a fourier transform infrared spectroscopy (FTIR) (TENSOR27).

### 2.3. TENG Output Measurement

The output performances of the NR-based TENGs were tested by measuring electrical output voltage and current using a vertical contact-separation mode with a single electrode configuration. A PTFE sheet was used as a contact triboelectric material. The voltage and current output signals were acquired under the mechanical impact force of 10 N with impact frequency of 5 Hz using an oscilloscope (Tektronix DPO2002B, Tektronix China Ltd, Shang Hai, China) and a digital ammeter (Kiethley DMM6500, Tektronix China Ltd, Shang Hai, China), respectively.

## 3. Results

**Figure 1:**
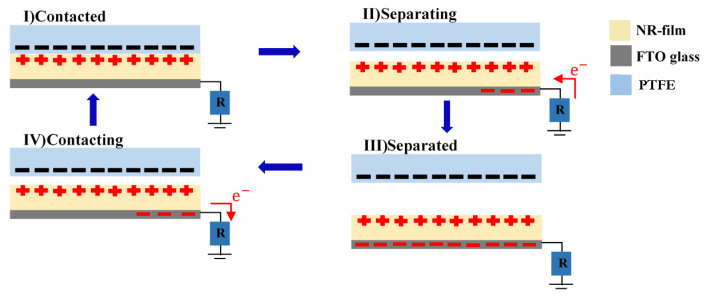
Schematic diagram of the device configuration for measuring energy conversion performance with working mechanism of the fabricated TENG under a vertical-contact separation mode with single electrode configuration.

The electrical output of the NR-TiO_2_ at 0.1–0.5%wt were measured under a vertical contact-separation mode with a single electrode configuration, as presented in Figure 1. PTFE was used as a contact material with negative triboelectric polarity. The electrical voltage and current were generated by the physical contact-separation of the NR-TiO_2_ film and PTFE surfaces. When the surfaces are in contact, the electrification effect causes electrons to be transferred between the two materials, resulting in the formation of positive and negative charges on surfaces of NR-TiO_2_ film and PTFE, respectively. When the two surfaces were separated, electrostatic induction of triboelectric charges allowed free electrons in the electrical contact to flow, neutralizing triboelectric charges on the surface. Under the repeated contact-separation, the alternative current was generated.

**Figure 2:**
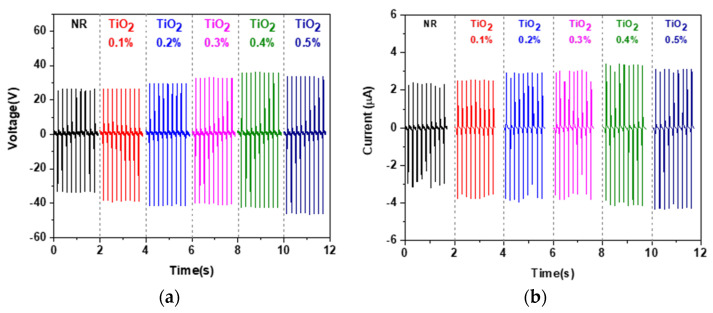
(**a**) Electrical output voltage and (**b**) current of the NR-TiO_2_ 0.1–0.5%wt.

The generated voltage and current of the fabricated NR-TiO_2_ TENGs using as-received TiO_2_ powders are presented in Figure 2a,b, respectively. The electrical outputs of the NR-TiO_2_ TENGs with as-received TiO_2_ increased with increasing TiO_2_ content and were at the highest in the NR-TiO_2_ 0.5%wt TENG, which were 78.4 V and 7.0 µA, respectively. However, the improvement of electrical output was not significant. It was suspected that the as-received TiO_2_ nanoparticles were agglomerated, giving rise to the poor dispersion in the NR matrix. 

**Figure 3:**
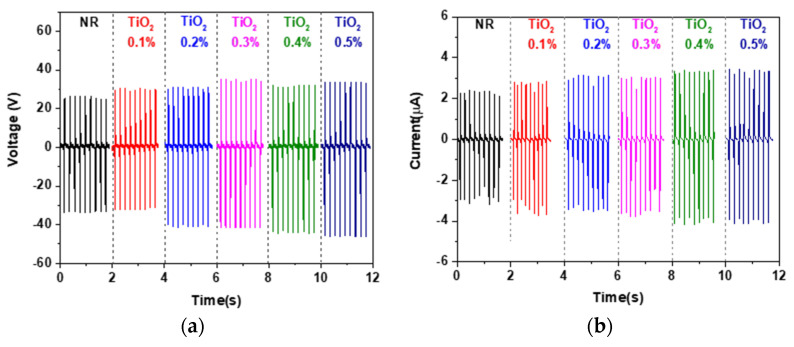

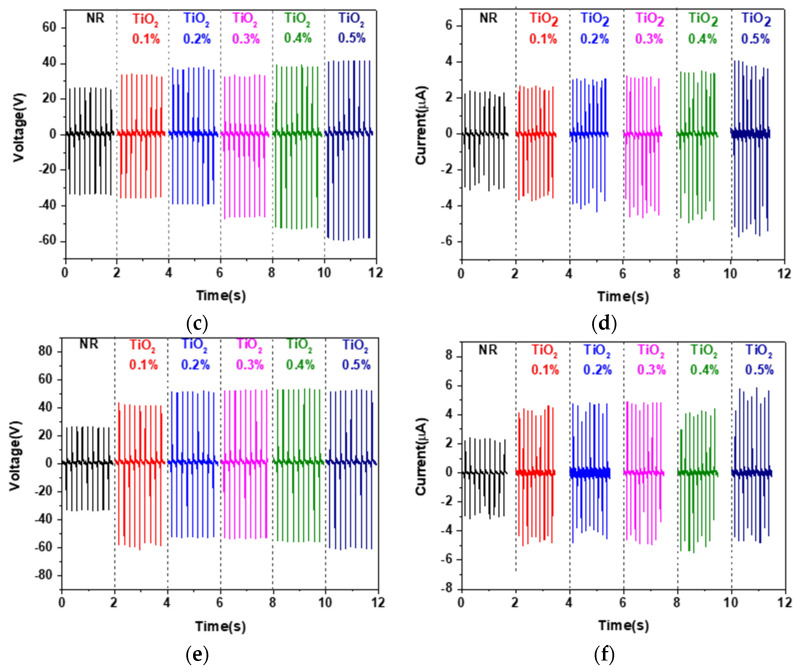
Electrical voltage and current of (**a**,**b**) NR-TiO_2_-B6h TENG, (**c**,**d**) NR-TiO_2_-B12h, (**e**,**f**) NR-TiO_2_-B24h 0.1–0.5%.

**Figure 4:**
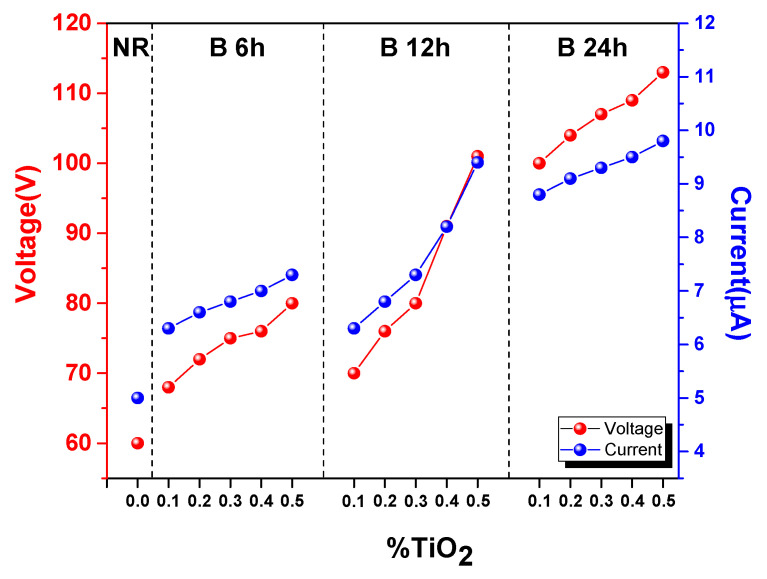
Electrical voltage and current of NR TENG and the NR-TiO_2_-composite TENGs fabricated from ball-milled TiO_2_ at 6, 12, and 24 h at 0.1–0.5%wt concentration.

In order to improve the dispersion in the NR matrix, TiO_2_ nanoparticles were ball-milled for 6, 12, and 24 h periods, prior to mixing with the NR latex to form composite materials. The ball-milled TiO_2_ at 0.1–0.5%wt (same as above experiment) were added to NR latex. Electrical output voltage and current of all the ball-milled NR-TiO_2_ TENG are displayed in Figure 3 and are summarized in Figure 4. It was found that ball-milled TiO_2_ helped to improve the electrical outputs of NR-TiO_2_ TENG, which increased with ball-milling time. The dependence of electrical output on TiO_2_ concentration of the ball-milled TiO_2_ TENG exhibited the same trend, as electrical output increased with increasing TiO_2_ concentration. The addition of the 24-h-ball-milled TiO_2_ nanoparticles into NR significantly improved TENG performance, and the highest output voltage of 113 V and current of 9.8 µA was achieved from the NR-TiO_2_-B24h-0.5%wt TENG. The enhancement of TENG performance was attributed to the disintegration of TiO_2_ nanoparticles at long ball-milling times, producing the well-dispersion in the NR polymer matrix. The role of TiO_2_ nanoparticles on TENG performance will be further discussed in the dielectric properties in the following section.

**Figure 5:**
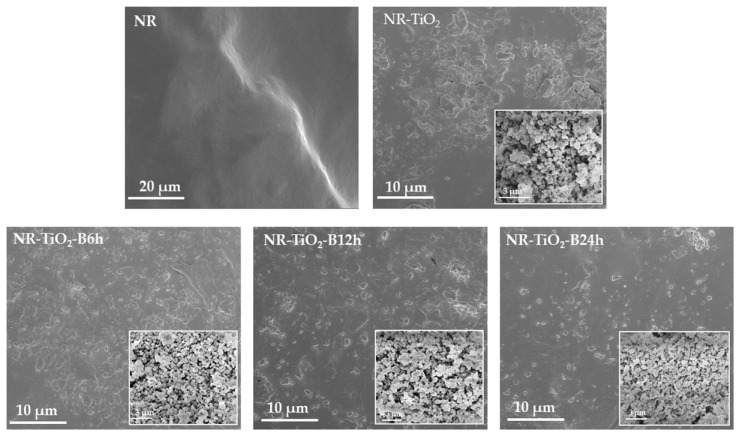
SEM images of the pristine NR film, NR-TiO_2_, NR-TiO_2_-B6h, NR-TiO_2_-B12h and NR-TiO_2_-B24h composite films at 0.5%wt with the insets of their TiO_2_ particles fillers.

**Figure 6:**
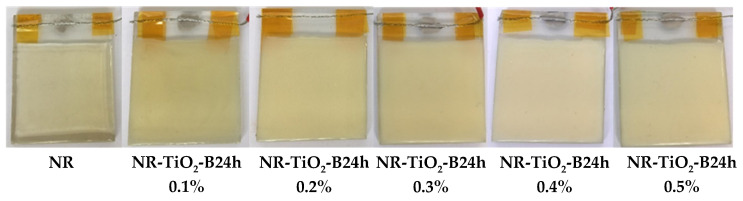
Digital photograph of the NR and NR-TiO_2_-B24h 0.1–0.5%wt.

The SEM images of the plain NR film, NR-TiO_2_, NR-TiO_2_-B6h, NR-TiO_2_-B12h, and NR-TiO_2_-B24h composite films at TiO_2_ 0.5%wt are displayed with the insets of their TiO_2_ nanoparticle fillers in Figure 5. Clearly, the dispersion of TiO_2_ without the ball-milling treatment was poor, as evidenced by the large agglomeration size of particles observed in SEM images of TiO_2_ powders and NR composite film. The agglomeration of TiO_2_ nanoparticles was less observed in the ball-milled TiO_2_ powders, which was reduced with increasing ball-milling times, contributing to the better dispersion in the NR composite films accordingly. The physical appearances of the NR and NR-TiO_2_-B24h 0.1–0.5%wt composite films are presented in Figure 6. The transparency of the pure NR film decreased as the TiO_2_ content increased. 

**Figure 7:**
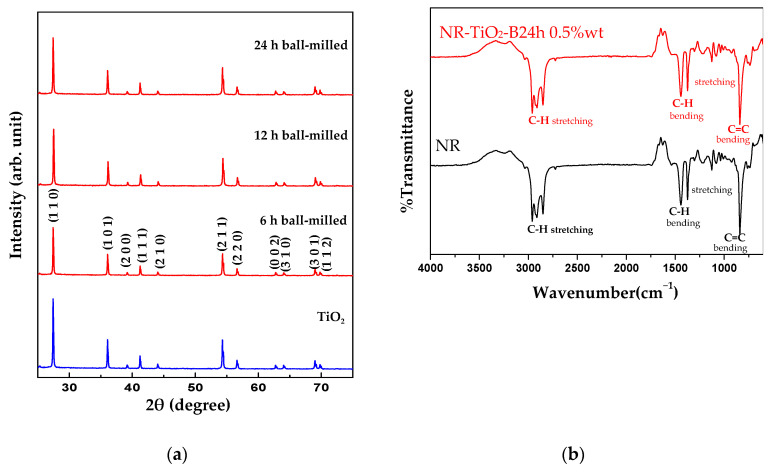
(**a**) XRD spectra of as-received TiO_2_ and ball-milled TiO_2_ at 6, 12, and 24 h. (**b**) FTIR spectra of the NR film and NR-TiO_2_-B24h 0.5%wt composite film.

The rutile phase of as-received and ball-milled TiO_2_ at 6, 12, and 24 h samples were confirmed by the XRD patterns as shown in Figure 7a (JCPDS No. 21–1276). This suggested that the ball-milling process did not change the crystal structure of the TiO_2_ nanoparticles. In this study, ball-milling was employed to break up the agglomerated particles and rutile phase is the most stable structure of TiO_2_; therefore, it should not cause the microstructural change of the particles. FTIR analysis of the NR and NR-TiO_2_-B24h 0.5%wt was performed and presented in Figure 7b. FTIR spectra of the NR and NR-TiO_2_-B24h 0.5%wt film are relatively similar, consisting of C-H stretching at 2850–2960 cm^−1^ and 1300–1400 cm^−1^ and C=C stretching at 839 cm^−1^ of polyisoprene molecules [30], and some C-O hydroxyl groups from non-rubber components in latex such as inorganic substances, proteins, phospholipids, carbohydrates, and fatty acids [16,31]. This suggested that no chemical bond was formed between TiO_2_ and NR polymer.

TENG electrical output is essentially a function of triboelectric charge density (*σ*) that forms upon contact electrification. For the contact mode TENG under open-circuit (OC) condition, the open-circuit voltage (*V_oc_*) is expressed by [32]

(1) $$V_{oc}=\frac{\sigma x\left(t\right)}{\varepsilon_{0}}$$

and short circuit current (*I_sc_*) is given by

(2) $$I_{sc}=\frac{S\sigma d_{0}v\left(t\right)}{{\left(d_{0}+x\left(t\right)\right)}^{2}}$$

where *ε*_0_, *S*, *d*_0_, *x*(*t*), and *v(t)* are electrical permittivity of free space, contact area size, effective thickness constant, separation distance, and contact electrode velocity, respectively. 

Triboelectric charge density depends on the material contact couple, contact area, as well as the charge storing ability of the surface. In the latter case, it refers to the dielectric constant of the material. For a contact-separation mode TENG which can be considered by a capacitive model, triboelectric charge is proportional to the capacitance of the device, which is given by [18]

(3) $$C=\frac{\varepsilon_{0}\varepsilon_{r}S}{d}$$

where *ε_r_* is dielectric constant and *d* is thickness of triboelectric material.

**Figure 8:**
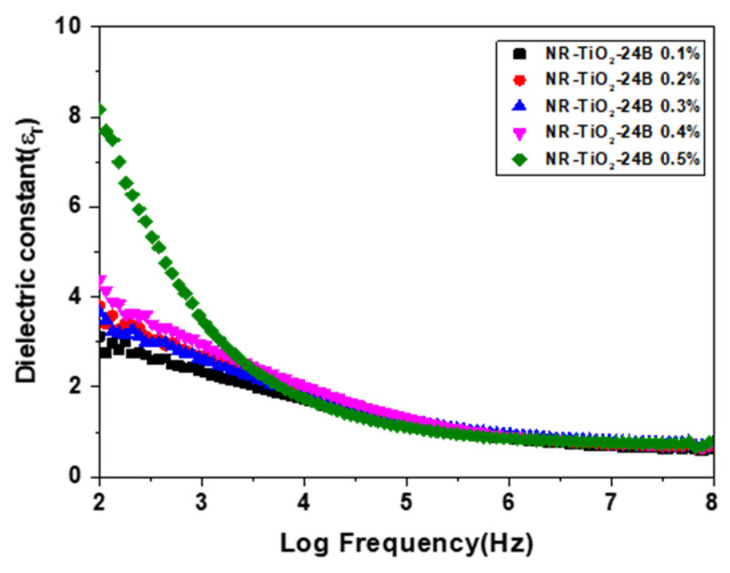
Dielectric constant of the NR-TiO_2_-B24h 0.1–0.5%wt.

Dielectric constants of the NR-TiO_2_-B24h 0.1–0.5%wt films measured at the frequencies ranging from 10^2^–10^8^ Hz is presented in Figure 8. The dielectric constant at 1 kHz of the NR-TiO_2_-B24h was found to increase with TiO_2_ concentration. The improvement of dielectric constant in the NR-TiO_2_-B24h films with increasing TiO_2_ concentration was ascribed to the fact that TiO_2_ has a greater dielectric constant than NR. The addition of increasing TiO_2_ filler concentration to NR polymer matrix gave rise to the increasing dielectric constant of the composites. The dielectric constant contributed to the charge capacitance at the surfaces of triboelectric materials, which intensified triboelectric charges that attributed to the increased electrical output of the TENG. 

**Figure 9:**
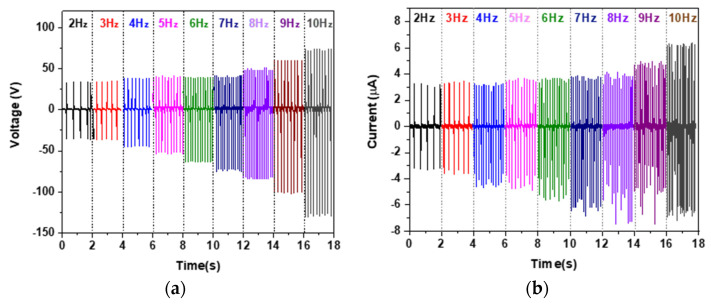
The frequency dependence of (**a**) electrical voltage and (**b**) current of the NR-TiO_2_-B24h 0.5%wt TENG.

The dependence of the output performance on the contact-separation frequency were also studied. The voltage and current outputs of the NR-TiO_2_-B24h 0.5%wt TENG were measured at operation frequencies ranging from 2–10 Hz, as presented in Figure 9a,b, respectively. It was found that electrical outputs depended on working frequency, and that the highest peak-to-peak voltage and current were 204 V and 13 µA, respectively, at a working frequency of 10 Hz. The increased electrical output was caused by charge retention on the surface due to a short contact-separation cycle at high frequencies.

**Figure 10:**
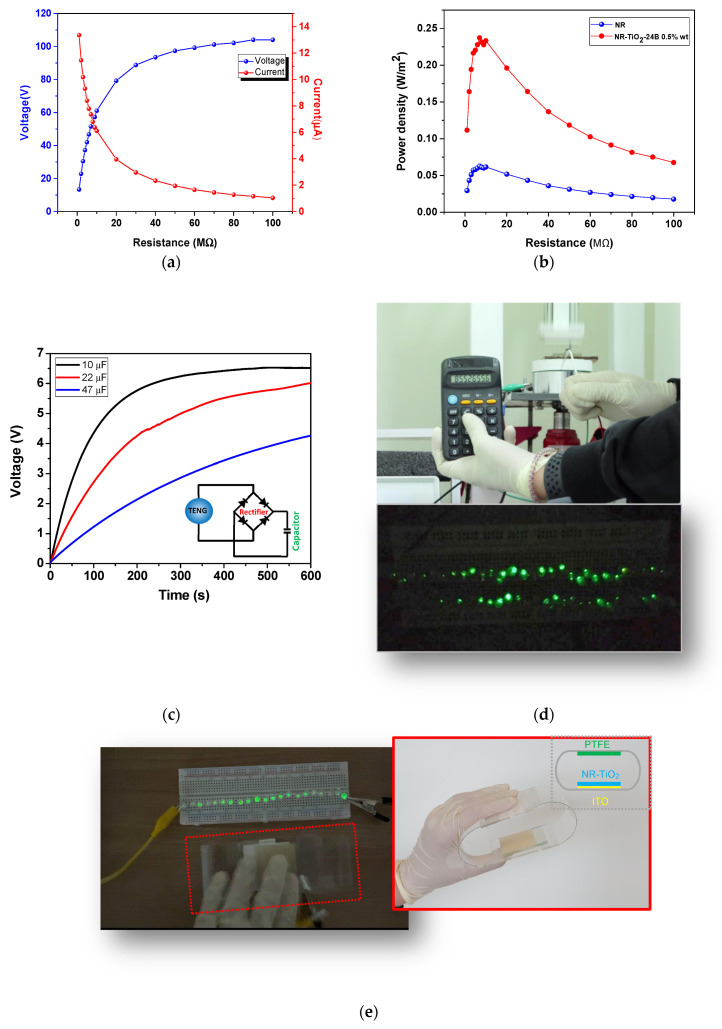
(**a**) The plot of voltage and current output versus load resistance and (**b**) Power density of the NR-TiO_2_-B24h 0.5%wt TENG compared to NR TENG. (**c**) Voltage profile of TENG to charge up the 10, 22, and 47 µF capacitors. (**d**) The demonstrations of TENG to operate a portable calculator (top) and to light up 60 green LEDs (bottom). (**e**) The fabricated TENG device able to light up 21 green LEDs by hand pressing with the inset showing a schematic diagram of device components.

The delivered power density of the NR-TiO_2_ TENG was also studied by measuring voltage and current at different load resistances ranging from 1–100 MΩ. The plot of voltage and current versus load resistances is shown in Figure 10a. The working power density of 200–237 mW/m^2^ was achieved at load resistances ranging from 3–20 MΩ and the maximum power density of 237 mW/m^2^ was achieved at a matched load resistance of 7 MΩ (Figure 10b), which was 3.6 times larger than that of pristine NR TENG (66 mW/m^2^). This electrical output was enough to charge up a 10, 22, and 47 µF capacitors, as presented in a voltage profile in Figure 10c, and was able to charge a 99 µF to operate a portable calculator and light up 60 green LEDs, as demonstrated in Figure 10d and Video S1 in the Supplementary Materials. In addition, a TENG device was fabricated which was able to light up 21 green LEDs by hand pressing, as demonstrated with the inset showing the schematic diagram of device components in Figure 10e.

## 4. Discussion

In the present work, the development of NR TENG with enhanced performance was demonstrated by the incorporation of TiO_2_ nanoparticles. The improved TENG performance was attributed to the enhanced triboelectric charge density by enhancing the dielectric constant of materials, as discussed in the previous section. TiO_2_ nanoparticles were employed as an effective filler for improving dielectric constant of NR composite film due to the high dielectric constant of TiO_2_. However, the agglomeration of nanoparticles suppressed the dispersion of nanoparticles in the NR matrix leading to an insignificant improvement of TENG performance, as presented in Figure 2. In this work, the simple and efficient approach to reduce the agglomeration of TiO_2_ nanoparticles using ball-milling was proposed. TiO_2_ nanoparticles were ball-milled prior to mixing with NR latex, which was found to effectively reduce the agglomeration of nanoparticles, as evidenced by SEM images (Figure 5), which then consequently produced the well-dispersion of TiO_2_ in NR-TiO_2_ composite films. In this work, the milling time of 24 h was found to efficiently reduce the agglomeration and produce the uniformly dispersed TiO_2_ in the NR films. The power output enhancement of the NR-TiO_2_-B24h was attributed to the improved dielectric constant due to the good dispersion of TiO_2_ nanoparticles. This suggested that ball-milling was an effective treatment to alleviate the agglomeration of TiO_2_ nanoparticles, which magnified the TENG electrical output to about 1.5 times higher than the untreated TiO_2_ composite TENG.

Comparing to other previous reports, the fabricated TENG showed a superior performance than the PDMS-Kapton-implanted TENG with a power density of 8.44 mW/m^2^ [33], the 2D woven wearable TENG fabricated from nylon and polyester threads with a power density of 2.33 mW/m^2^ [34], and approaching a propeller TENG made of PTFE and Al triboelectric materials with a power density of 283.95 mW/m^2^ [35]. In addition, comparing to the NR-based TENG, the NR-TiO_2_ TENG exhibited the comparable output power to the NR-Ag TENG in our previous report which was 262.4 mW/m^2^ [36]. The slightly lower TENG electrical output of the NR-TiO_2_ composite than that of the NR-Ag one was attributed to the lower dielectric constant of the NR-TiO_2_. The conductive Ag filler produced stronger interfacial polarization than the TiO_2_ semiconductor filler in the NR insulating matrix [37]. Therefore, the main contribution for the improved dielectric constant of NR-TiO_2_ was from the intrinsic dielectric property of TiO_2_, as described earlier. 

One of the most attractive aspects for employing NR as triboelectric material is the ability to scale up the production for large-area energy harvesting, owing to its low fabrication cost and feasibility to form composite with other materials. Comparing to other triboelectric polymers mentioned above, the costs of NR and TiO_2_ are much lower. In addition, the fabrication process of NR-TiO_2_ composite in the present work is straightforward, low cost and effective, which is promising for the development of large-scale energy harvesting device.

## 5. Conclusions

The NR-TiO_2_ TENG for harvesting mechanical energy into electricity was successfully fabricated. The addition of rutile TiO_2_ nanoparticles at 0.5%wt of NR latex to form NR-TiO_2_ composite was found to enhance energy conversion efficiency of the TENG. The modification of TiO_2_ by the ball-milling technique for 24 h prior to mix with NR materials was found to effectively disintegrate TiO_2_ nanoparticles which consequently helped the dispersion of the nanoparticle fillers in the polymer matrix. Owing to the high dielectric constant of TiO_2_ fillers, the dielectric constant of the NR-TiO_2_-B24h film increased with increasing TiO_2_ concentration. The NR-TiO_2_-B24h film with improved dielectric constant attributed to the enhancement of TENG electrical output with the highest power density of 237 mW/m^2^. This work showed the potential applications of NR-TiO_2_ TENG as an environmentally friendly power source for portable electronic devices.

## Data Availability

The data presented in this study are available in the article.

## Supplementary Materials

The following are available online at https://www.mdpi.com/article/10.3390/polym13132213/s1, Video S1: The demonstrations of TENG to operate a portable calculator.
